# Screening of Human Proteins for Fluoride and Aluminum Binding

**DOI:** 10.6026/97320630014068

**Published:** 2018-02-28

**Authors:** Md. Kamrul Hasan, Saruar Alam, Jovan Mirkovic, Md. Faruk Hossain

**Affiliations:** 1 Department of Biochemistry and Molecular Biology, University of Dhaka, Dhaka, Bangladesh;; 2 Department of Biological Sciences, St. John's University, Queens, New York 11439

**Keywords:** Fluoride, Aluminium, Neurodegenerative disorders, Human Health

## Abstract

Previous studies showed that prolonged exposure to fluoride (F-) and aluminum (Al3+) ions is associated with numerous diseases including neurological disorders. They don't have any known biological function. But they can bind with proteins that interact with ions similar to them. Such unwanted interactions affect the normal biological function of the target proteins, as well as their downstream protein-protein interactions. Several studies show the detrimental effects posed by them including Alzheimer's disease. However, their target proteins have never been reported. Here, we have screened for the human protein targets subjected to F- and Al3+ interactions by using data-driven prediction tools. We have identified 20 different proteins that directly bind with them (10 interact with fluoride and 10 with aluminum). In addition, protein-protein interaction has been explored to find the proteins that indirectly interact with F- and Al3+. We have found 86 indirect targets for F- and 90 for Al3+. Furthermore, 19 common protein targets have been identified, including proteins (9 out of 19) associated with neurodegenerative disorders. However, wet lab experiments are beyond our scopes to validate the binding networks. Additional studies must be warranted.

## Background

Fluorine (F) and aluminum (Al) are widely used in additives. Fluorine commonly appears as fluoride ion (F-) that reacts with other positively charged cations [1]. Sodium fluoride, fluorosilicic acid or sodium fluorosilicate, and sodium mono fluorophosphate are the common compounds of fluoride being utilized. Humans are exposed to sodium fluoride (NaF) from different sources including water, medicines, dental restorative materials, dental products (toothpaste and mouth rinses), pediatric supplements, beverages prepared with fluoridated water [2], tea, tobacco, pan masala (with tobacco and without tobacco) [3], and residues of pesticides, insecticides, and fertilizers [3]. Fluoride toothpaste is the most common source of fluoride ingestion into the human body. Research shows that 10% kids swallow toothpaste more than two fold the normal amount while brush their teeth [47].

Aluminium is the most abundant metal in nature having no known biological function [4]. It is an omnipresent element found in all types of products including corn, salt, herbs, spices, tea, yellow cheese, cosmetics, and aluminium ware and containers. Compounds of aluminium are used to purify drinking water and to treat wastewater plants [5]. Aluminium compounds are used in many medicines and pharmaceuticals (antacids, analgesics, antiperspirants). Presently, there is much concern about the presence of aluminium in drinking water [7, 8] and its employment in water treatment processes (as coagulant) and as metal in consumer products [5, 6]. In addition, aluminium can be leached from cooking vessels in case of storage of acidic foods [9].

There is evidence that fluoride and aluminum individually contribute to the development of different disorders. Fluoride causes detrimental effects on proteins expressed in soft tissues including the blood, brain, and liver [10] by penetrating the cell membrane [11]. Fluoride buildup in bone tissues disrupts the hematopoiesis in the bone marrow [12]. Numerous studies have demonstrated that high concentrations of aluminum are present in the brains of amyotrophic lateral sclerosis and senile dementia patients [13, 14]. The excess of both fluoride and aluminum may cause excitotoxicity through microglial activation and induce neurotoxicity [15]. The Blood Brain Barrier (BBB)-inhibits passive diffusion-is susceptible to fluoride and aluminum. Aluminum, as well as aluminum fluoride (AlF3), can react with oxygen to form aluminum oxide (Al2O3), which is found in significant amount in the brain specimen of Alzheimer patients [16]. AlF3 also substantiates osteoporosis, osteomalacia, spontaneous bone fractures, and dementia [17]. Identified protein targets for fluoride and aluminum in this analysis are involved in various important biological functions, for instance, PPP3CC has a role in the apoptotic signaling pathway, FGF12 is involved in nervous system development, etc. Data-driven prediction tools are used to screen for the targets. However, In vitro experiments are warranted to validate the finding in this study.

## Methodology

### Network analysis/ retrieval

The first step in the prediction of the potential protein targets of fluoride and aluminum is to find the network of interacting proteins with F- and Al3+ ions in human. We have used the STITCH 5.0 (Search Tool for Interacting Chemicals) (http://stitch.embl.de/) web server to find the protein targets. The STITCH database aims to incorporate dispersed databases, texts, and prediction methods into a single, easy-to-use resource. The STITCH offers a comprehensive overview of chemical and protein interaction network for numerous organisms including binding affinities between them. The predicted interaction is global for an individual organism and does not distinguish tissue specificity [18]. Target proteins from the STITCH were then subjected to the STRING (Search Tool for the Retrieval of Interacting Genes/Proteins) analysis to identify protein-protein association network. The STRING 10.5 (https://stringdb.org/) database goals to combine physical and functional interactions between proteins from a diverse source, including literature mining, experimental data, and computational prediction [19].

### Retrieving the protein sequences

The protein sequences identified with the STRING 10.5 analysis were downloaded in FASTA format from NCBI protein database (http://www.ncbi.nlm.nih.gov/protein). Two FASTA files were downloaded, containing 86 and 90 proteins for fluoride and aluminum, respectively. Additionally, numerous data-driven tools further analyzed these proteins.

### Gene ontology analysis

Gene ontology (GO) of the retrieved protein sequences was analyzed by Blast 2 GO software (https://www.blast2go.com/) [20]. This tool provides a high-throughput functional annotation for gene product properties [21]. We have utilized the aforementioned tool to identify the association between the target proteins and with the ions in the biological processes, molecular function, and cellular component of the human.

### Identficication of common proteins

The Venny 2.1 (http://bioinfogp.cnb.csic.es/tools/venny/) web server [22] has been used to identify 19 common protein targets from the list of proteins mentioned above.

### Analysis of expression of common proteins

The organs expressing the 19 common genes and their expression level have been determined from the NCBI (https://www.ncbi.nlm.nih.gov/gene/) database that uses RPKM (Reads Per Kilo-base per Million mapped reads) to quantify gene expression level.

## Results

### Interaction prediction

**Figure 1:**
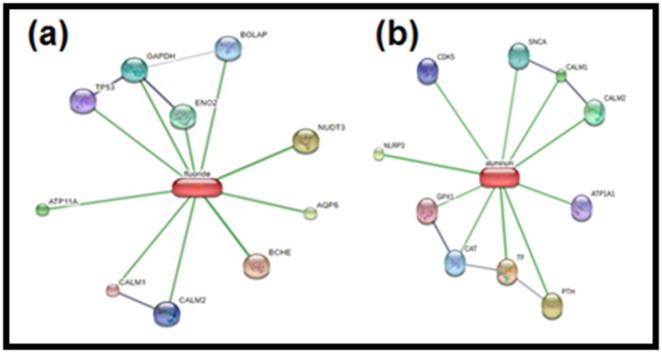
The network of interactions of (a) fluoride and (b) aluminum with protein targets predicted by STITCH 5.0. Fluoride and aluminum are represented as pill-shaped nodes, while proteins are shown as spheres. Nodes that are associated with each other are linked by an edge. The line's length refers to the binding affinities with each other. Greater affinity means shorter edge between the chemicals and the proteins and vice versa.

The STITCH 5.0 predicts the association for ion-protein network and their binding affinities. The STITCH 5.0 has predicted 20 human proteins for fluoride and aluminum interaction (10 proteins for each) (Figure 1). The STRING 10.5 predicts the network for protein-protein interaction. The STRING 10.5 has found 86 human proteins that interact with proteins predicted by the STITCH for fluoride and 90 for aluminum (Table 1 and 2).

### Pathway detection

**Figure 2:**
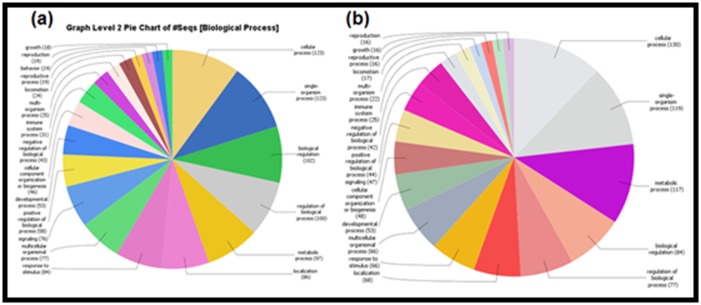
Pie graphs demonstrating the GO annotations against the direct protein targets of (a) fluoride and (b) aluminum. The diagrams represent the involvement of the proteins in the biological processes in human. The numbers in the parentheses represent how many GO annotations are present in each different biological process. Results are based on the Blast2GO data mining.

**Figure 3:**
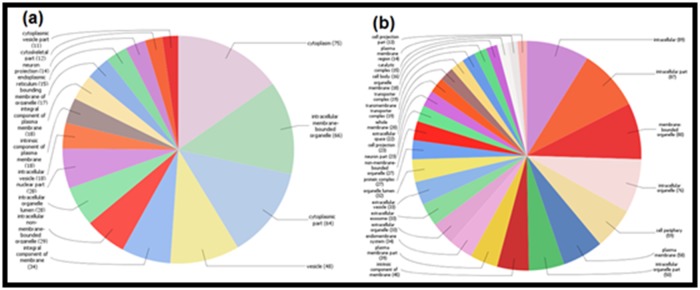
Pie diagrams demonstrating the GO annotations against the direct protein targets of (a) fluoride and (b) aluminum. The charts represent the association of the proteins in the molecular functions in human. The numbers in the parentheses represent how many GO annotations are present in each different molecular function. Results are based on the Blast2GO data mining.

**Figure 4:**
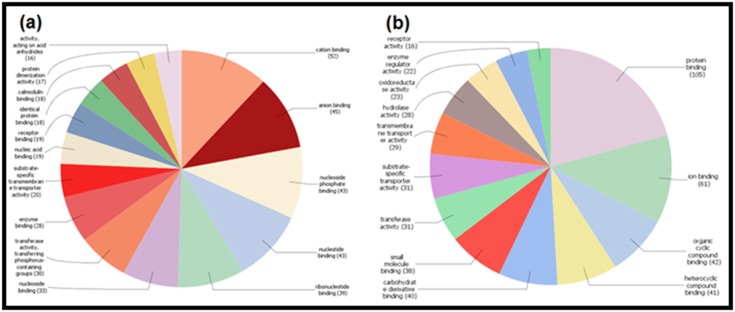
Pie charts demonstrating the GO annotations against the direct protein targets of (a) fluoride and (b) aluminum. The diagrams represent the involvement of the proteins in the cellular components in human. The numbers in the parentheses represent how many GO annotations are present in each different cellular component. Results are based on the Blast2GO data mining.

Through the Blast 2 GO software, we executed a series of steps for a list of target protein sequences of fluoride and aluminum, including protein Blast, mapping, and scanning motifs by Inter Pro Scan. The Gene Ontology (GO) has been performed to annotate the biological processes, molecular function, and cellular component for all the proteins found by the STITCH 5.0 and STRING 10.5 (Figures 2, 3, 4), and for 19 common proteins detected by the Venny web server (Figure 6).

### Common Targets Determination

**Figure 5:**
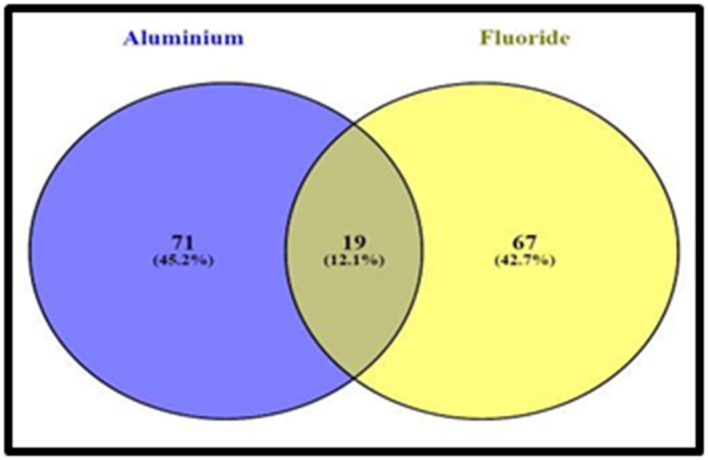
The common targets of fluoride and aluminum. The number in the Venn diagram (overlapping portion) represents the common targets. Analyzed by Venny web server.

**Figure 6:**
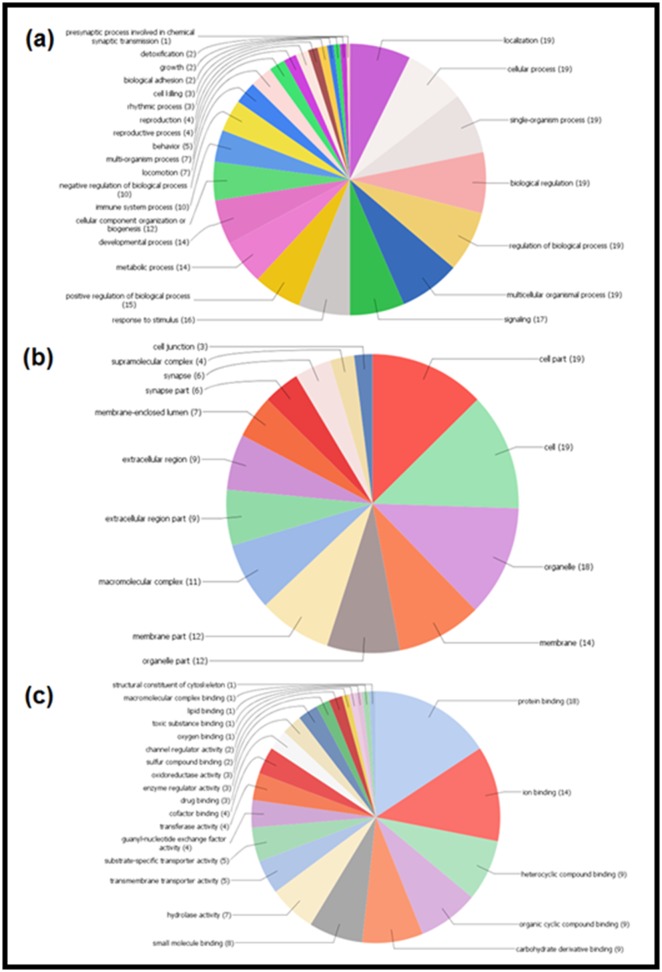
Pie diagrams demonstrating the GO annotations against the common protein targets of fluoride and aluminum. The charts represent the association of the proteins in the (a) biological processes, (b) cellular components, and (c) molecular functions in human. The numbers in the parentheses represent how many GO annotations are present in each different cellular processes. Results are based on the Blast2GO data mining.

The Venny 2.1 web server has been used to detect the 19 common protein targets of fluoride and aluminum (Table 3 and Figure 5).

## Discussion

Our findings suggest that fluoride and aluminum both have direct (ion-protein interaction) and indirect (protein-protein interaction) protein targets in the human. They directly bind with 20 proteins (both have 10 targets each), while the indirect binding network has 86 and 90 proteins for fluoride and aluminum, respectively. They have different binding affinities with their target, for instance, fluoride has a strong binding affinity with ENO2 compared to GAPDH and aluminum can strongly bind with GPX1 compared to SNCA. The GO annotation for the identified proteins demonstrates their association with numerous biological processes, cellular components, and molecular functions in the human. Both the ions have 19 common protein targets. We have further analyzed their expression site and expression level. RPKM data shows different expression level of the common target proteins of fluoride and aluminum in the human, for example, ALB (albumin) is highly expressed in the liver and SCN10A (Sodium Channel 10 Alpha subunit) is barely expressed in the testis. Most of the common target proteins are expressed in the brain. Fluoride and aluminum could have detrimental effects on many organs, including the spleen, liver, adrenal and prostate glands, small intestine, heart, and primarily the brain. The accumulation of aluminum-fluoride (AlF3) complex in the brain causes prolonged neurotoxicity, synaptic loss, and neurodegeneration [44]. AlF3 complex is able to impair the energy-producing enzymes; free radical balances, lipid peroxidation, and DNA repair mechanism [45, 46]. The target proteins are involved in different important functions, including programmed cell death, nervous system development, osmotic blood pressure regulation, action potential generation, etc. More importantly, most of the target proteins are brain-specific; therefore, chronic exposure to these ions could lead to the development of neurological disorders. One recent study showed that Al3+ could play a vital role as a mediator in the formation of fibrillary amyloid plaques in Alzheimer's disease [48].

Here, we find that APP or Amyloid beta (A4) precursor protein can interact with F- and Al3+. APP is a single-pass transmembrane protein highly expressed in the brain and is a key factor in the development of Alzheimer's disease [46]. It is possible that other target proteins of F- and Al3+ are associated with different diseases. However, further studies must be warranted to validate the binding network and to distinguish the physiologically important and unimportant bindings.

## Conclusion

Effects of fluoride and aluminum on living organisms especially on the humans come to light through long-term exposure to these ions. To minimize the exposure to fluoride and aluminum, public awareness is a must, for instance, parents should pay close attention to their children while they brush their teeth. In addition, companies should make toothpaste tasteless. We should avoid boiling the water in aluminum vessels, preserving acidic fruits and vinegar in the aluminum containers, preparing acidic fruit juice in the aluminum coated juicer, and use of automatic coffee machines etc. [47] to lower human exposure. Moreover, public policy, rules, and regulation should be implemented to minimize the exposures.

## Conflict of Interest

The authors have declared that no competing interest exists.

## Figures and Tables

**Table 1:** List of all proteins that directly and indirectly interact with fluoride. Analyzed by STITCH 5.0 and STRING 10.5 tools.

STITCH	CALM1	CALM2	BCHE	AQP6	NUDT3	BGLAP	GAPDH	TP53	ENO2	ATP11A
STRING	MYLK	FGF12	COLQ	TOP2A	SOX30	GGCX	ALDOA	ATM	GPI	ATP7A
	NOS1	PPP3CA	DOK7	TOP2B	MYCBP2	FURIN	ALDOB	CDKN1A	PGAM2	PRDM10
	NOS2	CAMK2A	CHRNA4	GK	MGAM	SPP1	ALDOC	CDKN2A	PGAM4	WDTC1
	NOS3	CAMK2B	CHAT	GK5	RPS10	BMP2	PGK1	MDM2	PGAM1	ATP7B
	PPP3CA	NOS3	APOE	SHPK	AP1S2	RB1	TPI1	MDM4	GAPDH	ATP1B2
	PPP3CB	CAMK2G	F2	GPD2		TBP	BPGM	BAX	PGK1	ATP1A3
	PPP3CC	SPTAN1	ALB	GK2		RUNX2	PGK2	BCL2	PGK2	ATP6V1A
	CAMK2A	SCN10A	APP	AQP10		IBSP	ENO1	CREBP	PKM	DNAH8
	CAMK2B	SCN11A	AHSG	AQP11		ATF4	ENO2	CDK2	TPI1	MGAM
	CAMK2G	SCN5A	ADAMTS2	AQP12A		PTH	ENO3	TP53BP2	PKLR	ATP1B3

**Table 2:** List of all proteins that directly and indirectly interact with aluminum. Analyzed by STITCH 5.0 and STRING 10.5 tools.

STITCH	CALM1	CALM2	ATP1A1	PTH	TF	CAT	GPX1	NLRP3	CDK5	SNCA
STRING	MYLK	FGF12	FXYD4	PTH2	TFR2	ALDH3A2	GSTT2B	TXNIP	CABLES1	UCHL1
	NOS1	PPP3CA	FXYD6	CALCA	HFE	HAO1	GSTP1	BRE	MAPT	UBB
	NOS2	CAMK2A	FXYD7	AVP	ALB	HAO2	GSTT1	IL1B	DPYSL2	SIAH2
	NOS3	CAMK2B	FXYD2	SCT	CFTR	ALDH2	GSTM1	AIM2	CDK5R1	SNCAIP
	PPP3CA	NOS3	FXYD1	PTH1R	EGF	SOD2	GSR	NLRC4	PPP1R1B	PARK2
	PPP3CB	CAMK2G	FXYD3	PTH2R	EGFR	AKT1	GSS	PYCARD	CDK5R2	PARK7
	PPP3CC	SPTAN1	ATP1B1	SCTR	APOB	SOD3	HPGDS	CARD8	CDK5R1	SLC6A3
	CAMK2A	SCN10A	ATP1B2	NPSR1	RAB5A	SOD1	SOD1	CASP1	NDEL1	KLK6
	CAMK2B	SCN11A	ATP1B3	GCG	M6PR	PRDX1	SOD2	CASP5	CCNB1	APP
	CAMK2G	SCN5A	ATP1B4	PTHLH	TFRC	GSR	SOD3	NLRP1	CCNB2	FYN

**Table 3:** Expression site and expression level of common 19 target proteins on the basis of their RPKM

Protein	Organ	RPKM
MYLK	Prostate	187.535±50.564
NOS1	Brain	1.116±0.49
NOS2	Small intestine	10.258±12.184
NOS3	Spleen	28.438±5.532
PPP3CA	Brain	54.022±15.795
PPP3CB	Brain	40.219±13.441
PPP3CC	Testis	12.255±3.702
CAMK2A	Brain	112.156±36.88
CAMK2B	Brain	44.021±23.5
CAMK2G	Brain	28.686±4.302
FGF12	Heart	20.774±12.935
SPTAN1	Brain	87.712±17.558
SCN10A	Testis	0.0088±0.054
SCN11A	Spleen	1.852±0.559
SCN5A	Heart	17.627±3.827
ATP1B2	Brain	126.89±28.341
ATP1B3	Adrenal	108.403±49.947
ALB	Liver	41385.4±9345.518
APP	Brain	395.222±72.782

**Table 4:** A short description of the common binding targets of F- and Al with their normal physiological functions.

Name	Short description	Functions	Reference
MYLK	Myosin light chain kinase	Implicated in smooth muscle contraction via phosphorylation of myosin light chains. Involved in inflammatory response and cell migration. Pseudogene of MYLK found having carcinogenic effect.	[23]
NOS1	Nitric oxide synthase 1 (neuronal)	Process involved in neurotransmitter biosynthesis and secretion. Polymorphism in this gene has association with neurological disorder and asthma.	[24]
NOS2	Nitric oxide synthase 2 (inducible)	Nitric oxide-mediated cell signaling pathway and variation found in asthma patients.	[25]
NOS3	Nitric oxide synthase 3 (endothelial cell)	Vascular smooth muscle relaxation through a cGMP-mediated signal transduction pathway has association with variation in this gene has association with asthma and Alzheimer disease.	[26]
PPP3CA	Protein phosphatase 3, catalytic subunit, alpha isozyme	Calcium ion transport, calcinurin-NFAT signaling cascade	[27]
PPP3CB	Protein phosphatase 3, catalytic subunit, beta isozyme	Calcineurin-NFTA signaling cascade, regulate synaptic spacticity has an association with schizophrenia.	[28]
PPP3CC	Protein phosphatase 3, catalytic subunit, gamma isozyme	Role in apoptotic signaling pathway has association with schizophrenia.	[29]
CAMK2A	Calcium/calmodulin dependent protein kinase II alpha	Role in the regulation of synaptic plasticity. Mutation in this gene result in neurological disorder.	[30]
CAMK2B	Calcium/calmodulin-dependent protein kinase II beta	Regulation of calcium ion transport and synapse structural plasticity.	[31]
CAMK2G	Calcium/calmodulin-dependent protein kinase II gamma	Involved in sarcoplasmic reticulum Ca2+ transport in skeletal muscle and may function in dendritic spine, synapse formation, and neuronal plasticity.	[32]
FGF12	Fibroblast growth factor 12	Involved in nervous system development and function	[33]
SPTAN1	Spectrin, alpha, non-erythrocytic 1	Calcium-dependent movement of the cytoskeleton at the membrane	[34]
SCN10A	Sodium channel, voltage-gated, type X, alpha subunit	Mediates the voltage-dependent sodium ion permeability of excitable membranes	[35]
SCN11A	Sodium channel, voltage-gated, type XI, alpha subunit	Encodes Nav1.9, a voltage-gated sodium ion channel which functions as key relay stations for the electrical transmission of pain signals from the periphery to the central nervous system	[36]
SCN5A	Sodium channel, voltage-gated, type V, alpha subunit	Responsible for the initial upstroke of the action potential	[37]
ATP1B2	ATPase, Na+/K+ transporting, beta 2 polypeptide	Catalyzes the hydrolysis of ATP coupled with the exchange of Na+ and K+ ions across the plasma membrane	[38]
ATP1B3	ATPase, Na+/K+ transporting, beta 3 polypeptide	Catalyzes the hydrolysis of ATP coupled with the exchange of Na+ and K+ ions across the plasma membrane	[39]
ALB	Serum albumin, the main protein of plasma	Regulating the colloidal osmotic pressure of blood	[40]
APP	Amyloid beta (A4) precursor protein	Triggering caspase activation and degeneration of both neuronal cell bodies and axons	[41]
